# Status of selected biochemical and coagulation profiles and platelet count in malaria and malaria-*Schistosoma mansoni* co-infection among patients attending at Dembiya selected Health Institutions, Northwest Ethiopia

**DOI:** 10.1038/s41598-024-56529-w

**Published:** 2024-03-13

**Authors:** Wagaw Abebe, Zelalem Asmare, Addis Wondmagegn, Mulat Awoke, Aderajew Adgo, Adane Derso, Wossenseged Lemma

**Affiliations:** 1 Department of Medical Laboratory Sciences, College of Health Sciences, Woldia University, Woldia, Ethiopia; 2 Department of Nursing, College of Health Sciences, Woldia University, Woldia, Ethiopia; 3 Department of Biotechnology, College of Natural and Computational Science, Woldia University, Woldia, Ethiopia; 4 Department of Medical Parasitology, School of Biomedical and Laboratory Sciences, College of Medicine and Health Sciences, University of Gondar, Gondar, Ethiopia

**Keywords:** Biochemical profile, Coagulation profile, Malaria, *S. mansoni*, Co-infection, Dembiya, Ethiopia, Biochemistry, Microbiology

## Abstract

Malaria and schistosomiasis are infectious diseases that cause coagulation disorders, biochemical abnormalities, and thrombocytopenia. Malaria and *Schistosoma mansoni* co-infection cause exacerbations of health consequences and co-morbidities.This study aimed to compare the effect of malaria and *Schistosoma mansoni* co-infection and malaria infection on selected biochemical and coagulation profiles, and platelet count. An institutional-based comparative cross-sectional study was conducted from March 30 to August 10, 2022. A total of 70 individuals were enrolled in the study using a convenient sampling technique. Wet mount and Kato Katz techniques were conducted to detect *Schistosoma mansoni* in a stool sample. Blood films were prepared for the detection of *plasmodium*. The data was coded and entered into EpiData version 3.1 before being analyzed with SPSS version 25. An independent t test was used during data analysis. A P-value of less than 0.05 was considered statistically significant. The mean [SD] of alanine aminotransferase, aspartate aminotransferase, creatinine, total bilirubin, and direct bilirubin in the co-infected was higher than in malaria infected participants. However, the mean of total protein and glucose in co-infected was lower than in the malaria infected participants. The mean of prothrombin time, international normalization ratio, and activated partial thromboplastin time in co-infected was significantly higher, while the platelet count was lower compared to malaria infected participants. Biochemical and coagulation profiles, and platelet count status in co-infection were changed compared to malaria infected participants. Therefore, biochemical and coagulation profiles and platelet count tests should be used to monitor and manage co-infection related complications and to reduce co-infection associated morbidity and mortality.

## Introduction

Malaria is an infectious disease caused by protozoan parasites of the genus *Plasmodium* and transmitted by female *Anopheles* mosquitos^1^*. Plasmodium falciparum, Plasmodium vivax, Plasmodium malariae, Plasmodium ovale,* and *Plasmodium knowlesi* are the species that infect humans among the *plasmodium* species^2^. The majority of malaria infections are caused by *P. falciparum and P. vivax* in the world^3^. Based on the World Health Organization (WHO, 2020), malaria caused 627,000 deaths and 241,000,000 cases globally in 2020^4^. An increase in malaria cases from 245 million in 2020 to an approximated 247 million cases globally in 84 malaria-endemic countries in 2021, with the WHO African Region causing the majority of this increase^3^. It was anticipated that 619,000 people would die from malaria in 2021. Ninety six percent of malaria deaths occur on the African continent, and children under five continue to account for the majority of malaria fatalities. In low-income nations, malaria is common, especially in children, pregnant women, and other susceptible groups like migrants^3,5^. Over 100 million people live in Ethiopia, and 68% of them are thought to be at risk for the illness. The annual reported cases of malaria are approximately 2.9 million, with 4,782,000 related deaths. During epidemics, the rate of morbidity and mortality rises rapidly^6^.

Malaria parasites go through a hepatocyte developmental stage. Sporozoites produced from the salivary gland of a mosquito must effectively target and penetrate hepatocytes^7^. These parasites replicate in the red blood cells of their human host following an initial replication phase in the liver. These erythrocytes replication cycles cause the typical disease symptoms, including fever and anemia, and eventually lead to organ failure and patient death^8^. Sequestration of erythrocytes with mature forms of the parasite in the deep vascular beds of vital organs is the major pathologic hallmark of severe malaria. *Plasmodium falciparum* malaria frequently causes life-threatening complications such as cerebral malaria, renal failure, hepatic dysfunction, jaundice, abnormal bleeding, and severe anemia^9^.

Malaria causes blood coagulation disorders, which contribute to inflammation and organ failure^10^. This is due to the sequestration of parasitized red blood cells in the microcirculation, generation of cytokines, and activation of the coagulation system in malaria patients^11^. Hence, malaria parasite-infected red blood cells enhance tissue factor expression by endothelial cells and support the assembly of coagulation complexes, generation of thrombin, activation of platelets, generation of microthrombi, and activation of the intrinsic pathway of coagulation^12^.

This causes prolongation of prothrombin time (PT) and activated partial thromboplastin time (APTT) and disseminated intravascular coagulation^13^. Due to peripheral platelet consumption and destruction, malaria results thrombocytopenia. Immune complexes are comprised of malaria antigens and Immunoglobulin G (IgG) and Immunoglobulin M (IgM) antibodies that cause platelets to be sequestered by macrophages in the spleen. This causes a reduction of platelet lifespan during malaria infection^14^.

Furthermore, malaria induces biochemical changes within the host^11^. Sporozoites invade hepatocytes in the liver stage, causing organ congestion, sinusoidal blockage, and cellular inflammation. Hepatocyte changes can result in the leakage of parenchyma and membrane enzymes into the general circulation^15^. Due to this, malaria causes biochemical abnormalities such as high bilirubin, elevated aspartate aminotransferase^16^, elevated alanine aminotransferase (ALT), and high creatinine, which increase the risk of disease complications^17^.

Schistosomiasis is a parasitic disease caused by the genus *Schistosoma* of blood-dwelling trematodes. According to the global burden of disease study, schistosomes infect 252,000,000 people, 90% of whom reside in sub-Saharan Africa, and are estimated to have cost the world 3,300,000 disability-adjusted life years^18^. *Schistosoma mansoni*, *Schistosoma haematobium,* and *Schistosoma japonicum* are the species which infect human being among the *Schistosoma* species^19^. *Schistosoma* species which causes disease, where it resides, and the severity of infection all influence the clinical presentation and pathology of the schistosomiasis^20^. The mesenteric plexus is a habitat to *S. mansoni,* and leads to intestinal or hepatosplenic schistosomiasis, which affect the intestine, liver, and spleen^21^. *Schistosoma* pathogenesis is mainly associated with the host’s immune responses to *Schistosoma* egg antigens, which results in the formation of granulomas in the intestine and the liver where the eggs are trapped^22^. This egg-induced granulomas causes liver failure, which leads to protein synthesis impairment, and increment of ALT and AST levels^23^.

Schistosomiasis also has an impact on hematological profiles, either directly through the gut or indirectly by aggravating blood loss through feces by rupturing blood vessels with the help of the egg spine^24^. The existence of thrombocytopenia in schistosomiasis patients may result from the association of schistosomiasis with splenomegaly, which enhances platelet destruction and filtering by the spleen^24^. Moreover, schistosomiasis causes coagulation disorders due to decrease hepatic synthesis of coagulation proteins, as well as decrease clearance of activated forms associated with consumption of coagulation factors*.* Due to the activation of the coagulation system, patients with schistosomiasis have elevated levels of coagulation activation markers, prolonged PT, and APTT, as well as extensive fibrin deposition over hepatic egg granulomas^25^.

Malaria and schistosomiasis are among the parasitic infections that shares common transmission areas in various tropical regions. The co-infection of malaria and schistosomiasis is common in Africa. In Ethiopia, the co-infection rate of schistosomiasis and malaria was reported to be 15%, resulting in a higher incidence of anemia when compared to individuals with malaria only^26^. Also, co-infections of these parasites are prevalent as a result of geographical overlap between schistosomiasis and malaria, resulting in various forms of association, exacerbated health consequences, and co-morbidities^27^. Moreover, the co-infection has a significant impact on the regulation of inflammatory factors associated with the progression of these infections and their respective morbidity^28^.

Studying on malaria and *S. mansoni* co-infection effect on biochemical and coagulation profiles, and platelet count is important to reduce different problems which are related to those co-infection like impaired protein synthesis, liver fibrosis, and coagulation disorders. Also, in order to put better clinical management and control of malaria, especially in malaria and *Schistosoma* co-endemic areas, information on the effect of malaria and *S. mansoni* co-infection and malaria infection on biochemical and coagulation profiles, and platelet count is needed. Furthermore, a clinician can develop a dependable diagnosis and effective treatment interventions for patients with malaria and *S. mansoni* co-infection by having knowledge of changes in biochemical and coagulation profiles as well as platelet count. However, study on the effect of co-infection with malaria and *S. mansoni* on biochemical and coagulation profiles and platelet count is still limited. Therefore, the current study attempted to compare the effect of malaria and *S. mansoni* co-infection and malaria infection on selected biochemical and coagulation profiles, and platelet count.

## Methods and material

### Study area

The study was conducted at Dembiya Primary Hospital, Chuahit Health Center, and Abrija Health Center, which are located within the Central Gondar Administrative Zone, Amhara Regional State. Dembiya Primary Hospital, Chuahit Health Center, and Abrija Health Center are found in Dembiya district. Dembiya district is located in Northwest Ethiopia, 35 km from Gondar, town of Central Gondar Administrative Zone, 183 km from Bahir Dar, capital of Amhara Region State and 762 km from Addis Ababa, capital city of Ethiopia, between 12° 39' N and 37°09' E. The southern part of the district is bordered by Lake Tana.

Dembiya district has a total population of 326,686, of whom 162,477 were men and 164,209 were women in 2017. It is located on altitude of between 1500 and 2600 m above sea-level. Its average annual rainfall and average temperature range from 995 to 1175 mm and 21.5 °C, respectively. There is one governmental primary hospital, ten health centers, nine private clinics, and 49 Health posts providing health care service for Dembiya and its surrounding people^29^. Rivers within this district include Angereb and Derma. These rivers serve as sources of water for bathing, washing clothes, and other domestic and recreational purposes. They may contain the major sources of malaria and *S.mansoni* infections. As reported by the District Health Bureau, malaria and *S.mansoni* are endemic in the study area.

### Study design, period, and population

An institutional-based comparative cross-sectional study was conducted from March 30 to August 10/2022 at Dembiya Primary Hospital, Chuahit Health Center, and Abrija Health Center. The study populations were malaria and *S. mansoni* co-infected and malaria infected individuals. The study excluded pregnant women, people with multiple intestinal parasite infections, people receiving antiretroviral therapy, people with a history of chronic diseases like hypertension, cardiac disease, diabetes mellitus, chronic renal disease, and inherited bleeding disorders, people who were positive for hepatitis B and hepatitis C viruses, people who were taking anticoagulant therapy, smokers, and people who used alcohol excessively.

### Sample size determination and sampling technique

Sample size was determined based on rules of thumb that have been recommended by van Voorhis and Morgan, 30 study subjects per group are required to detect real differences, which lead to about 80% power^30^. Thus, 70 study participants (35 infected by both malaria and *S. mansoni* and 35 malaria infected participants, sex and age match control) were enrolled in the study. A convenient sampling technique was used to select study participants**.**

### Operational definition

The selected biochemical profiles were ALT, AST, creatinine, glucose, total bilirubin, direct bilirubin, and total protein. Similarly, the selected coagulation profiles were PT, INR, and APTT. The abnormalities of coagulation profiles and platelet count are, prolonged PT, PT > 16 Second, prolonged INR , INR > 1.1, prolonged APTT, APTT > 35 s, and low platelet count, platelet count < 150 × 103/µL^31^. Also, the normal value of ALT, AST, total bilirubin, direct bilirubin, creatinine, total protein and glucose were 0–41 U/L, 0–40 U/L, 0–1.2 mg/dl, 0–0.2 mg/dl, 0.7–1.2 mg/dl, 3.5–5.2 g/dl, and 74–109 mg/dl, respectively^32^.

### Data collection procedures

#### Questionnaire survey

Socio-demographic characteristics of study participant were collected using a semi-structured questionnaire prepared in Amharic language. The questionnaire was initially written in English language and translated to Amharic language. Socio-demographic data was collected by principal investigator (PI), trained Medical Laboratory Personnel and Nurses. Trained clinicians who work at Dembiya selected health institutions Outpatient department (OPD) assessed the clinical information and patient history. Following identifying individuals who were eligible for the study, then the volunteer study participants were linked to Medical Laboratory Personnel for blood and stool samples collection.

### Sample collection and laboratory examination

#### Microscopic detection of plasmodium

On a microscopic glass slide, 6 micro liter and 2 micro liter of capillary blood were placed separately for preparing thick and thin blood films, respectively. So blood films, both thick and thin, were prepared and air dried. Absolute methanol was used to fix thin blood films, and both films were stained for 10 min with a 10% Geimsa working solution.

An experienced malaria microscopist and PI read both thin and thick blood films with a 100 × objective lens and examine100 microscopic field’s to rule out the absence or presence of the malaria parasites. The discrepancy results were confirmed by another experienced malaria microscopist. Malaria parasitemia was determined in thick blood films along with 200 white blood cells. It was calculated by using the formula:Parasite/μL = Parasite counted/200WBC × Total white blood cells count. Parasitaemia was classified into three categories according to the number of parasites/μl of blood, low (< 1000), moderate (1000–9999) and high (≥ 10,000)^33^.

#### Microscopic detection of schistosome

Single stool specimen of about one gram was collected from each study participant. The sample was collected in a clean, dry, and leak-proof container with a unique identification number. Each stool specimen was first examined using the direct wet mount technique, and then Kato-Katz slides prepared on a template containing 41.7 mg of stool. Eggs counted for *S. mansoni* were recorded and later converted into eggs per gram (EPG) of stool, multiplying by a factor of 24^34^. Finally, infection intensity (light (1–99 EPG), moderate (100–399 EPG), and heavy (≥ 400 EPG)) was classified according to WHO criteria^35^.

#### Blood sample collection for biochemical and coagulation profiles and platelet count examination

Seven milli litre of venous blood was collected by blood collectors and PI using a sterile disposable plastic syringe after cleaning the venous puncture site with 70% alcohol. The collected blood sample was transferred into three test tubes. The first 2.7 ml of the collected blood sample was placed into 3.2 % sodium citrate anticoagulant test tube. For PT and APTT analysis, platelet-poor plasma was prepared by centrifuging at 1500 revolution per minute for 15 min^34^. Then plasma was separated and stored in Eppendorf tube at − 20 °C until processed. The coagulation profiles (PT, APTT, and INR) were done at Felege Hiwot Compressive Specialized Hospital Laboratory by using Semi-Auto Coagulation Analyzer (HumaClot Duo^Plus^ Human, Germany)^36^.

The 2 ml of blood was transferred into ethylene diamine tetra-acetic acid (EDTA) test tube for platelet count. Platelet count was determined using Fully Auto Hematology Analyzer^37^.

The remaining venous blood was transferred into nonanticoagulated tube and allowed to clot at bench top. Then, blood centrifuged at 2500 revolutions per minute for four minute and serum was separated and stored in Eppendorf tube at − 20 °C until processed. Then, serum was analyzed by using Fully Auto Chemistry Analyzer^38^ for serum level of ALT, AST, creatinine, glucose, total bilirubin, direct bilirubin, and total protein^38^.

##### Serological tests

Immune-chromatographic assay was used to determine hepatitis B virus and hepatitis C virus to exclude individuals who were positive for these diseases^34^.

##### Urine collection and HCG examination

Urine was collected from all women whose age is 15–49 years in the study using a clean urine cup and a urine human chorionic gonadotropin (HCG) test was performed for both the cases and the controls using a rapid chromatographic immunoassay test strip to exclude pregnant women^39^.

### Data quality control

Data collectors took appropriate training in order to maintain data quality. Quality control was performed by re-reading all slides by an expert laboratory technologist, to ensure the accuracy of the detection of *Plasmodium* and *Schistosoma* which was conducted by laboratory technologists. Standard operating procedures and manufacturer instructions were strictly followed throughout the procedures and all reagents were stored and prepared according to the manufacturer’s instructions.

### Data management and analysis

Data was coded and entered into the EpiData (v3.1) statistical software, and exported to the statistical package of social science (SPSS) version 25 for analysis. The homogeneity of variance was checked by using Levene’s statistics. The Skewness, kurtosis, and Shapiro–Wilk normality tests were used for checking the distribution of continuous variables, and it revealed that some data were normally distributed and some data were not normally distributed for each group. Independent t test Difference test was used for comparison of normally distributed biochemical and coagulation profiles, and platelet count between groups. For each group, the result was provided as the mean and SD for normally distributed data. A p-value of less than 0.05 was considered statistically significant in all statistical analyses.

### Ethics approval and consent to participate

This study was conducted after ethical approval was obtained from research and ethics committee of School of Biomedical and Laboratory Sciences, College of Medicine and Health Sciences, University of Gondar. Moreover, letter of support was submitted to the Dembiya Primary Hospital, Chuahit Health Center, and Abrija Health Center. Before starting the actual data collection, permission was obtained from the Hospital and Health Centers Chief Executive Officer or health facilities and the Administrator. Additionally, after explaining the purpose, benefits, and the possible risks of the study, written informed consent from the age of 16 and above and/or assent from those less than 16 years old study participants along with written informed consent from their respective parents/ caregiver/guardians was obtained. And also, written informed consent from illiterate study participants was obtained from their respective parents/guardians. All laboratory results were kept confidential. Since those were stored in a file using codes without study participants name. Apparently, those positive for parasites and with biochemical and coagulation profiles, and platelet count abnormality were linked to the hospital and health centers for appropriate treatment and management.

## Result

### Socio-demographic characteristics of study participants

A total of 70 study participants participated from Dembiya Primary Hospital [36 (51.4%)], Chuahit Health Center [18 (25.7%)], and Abrija Health Center [16 (22.9%)]. Participants from rural and urban were 38 (54.3%) and 32 (45.7%), respectively. Among 70 study participants, 35 were malaria and *S. mansoni* co-infected, and 35 were malaria infected participants. The prevalence of malaria and *S. mansoni* co-infection was higher in males (18, 51.4%) than females (17, 48.6%), and higher in the age group of 5–14 years (11, 31.4%) than those other age groups [15–24 years, 8 (22.9%), 25–34 years, 8 (22.9%), 35–44 years, 5 (14.3%), > 44 years, 3 (8.6%)] (Table 1).

**Table 1:** Socio-demographic characteristics of the study participants at Dembiya selected health institutions, 2022.

Socio-demographic characteristics	*S. mansoni* and malaria coinfection		Malaria	
	Frequency	%	Frequency	%
Sex				
Male	18	51.4	18	51.4
Female	17	48.6	17	48.6
Age				
5–14	11	31.4	7	20
15–24	8	22.9	11	31.4
25–34	8	22.9	9	25.7
35–44	5	14.3	2	5.7
> 44	3	8.6	6	17.1
Residence				
Urban	16	45.7	16	45.7
Rural	19	54.3	19	54.3
Occupation				
Government employee	5	14.3	5	14.3
Nongovernment employee	2	5.7	1	2.9
House wife	3	8.6	6	17.1
Student	13	37.1	12	34.3
Daily laborer	3	8.6	2	5.7
Farmer	7	20	8	22.9
Merchant	2	5.7	1	2.9
Educational status				
Illiterate	7	20	11	31.4
Can read and write	4	11.4	1	2.9
Primary school	14	40	10	28.6
Secondary school	3	8.6	8	22.9
College/university	1	2.9	2	5.7
Diploma and above	6	17.1	3	8.6
Family size				
1–3	12	34.3	18	51.4
4–6	7	20	13	37.1
7–9	11	31.4	3	8.6
> 9	5	14.3	1	2.9
Income				
500 Birr	2	5.7	4	11.4
501–1000	2	5.7	3	8.6
1001–2500	11	31.4	13	37.1
> 2500	20	57.1	15	42.9

### Intensity of malaria parasitaemia

The overall mean of malaria parasitemia on *S. mansoni* and malaria coinfected group was 10,891.9. The mean of parasitemia of malaria in males and females were 8784.2 and 14,931.8 in *S. mansoni* and malaria co-infected group, respectively. From a total of 35 *S. mansoni* and malaria coinfected group 4 (11.4%), 18 (51.4%), and 13 (37.1%) were due to low, moderate, and high malaria parasitemia infection, respectively. The overall mean of malaria parasitemia on malaria infected group was 8539.4. The mean of parasitemia of malaria in males and females were 7866.7 and 9251.8 malaria infected group, respectively. And also, from a total of 35 malaria infected group 3 (8.6%), 24 (68.6%), and 8 (22.9%) were due to low, moderate, and high malaria parasitemia infection, respectively (Table 2).

**Table 2:** Intensity of malaria parasitaemia among study participants at Dembiya selected health institutions, 2022.

Level of parasitaemia	*S. mansoni* and malaria coinfected	Malaria infected
Low	4 (11.4%)	3 (8.6%)
Moderate	18 (51.4%)	24 (68.6%)
High	13 (37.1%)	8 (22.9%)

### Biochemical profiles among study participants

The percentages of elevated ALT, AST, total bilirubin, direct bilirubin, and creatinine were higher in malaria and *S. mansoni* co-infected participants than in malaria infected participants. In addition, the percentages of lowered total protein and glucose were higher in malaria and *S. mansoni* co-infected participants than in malaria infected participants.

**Figure 1:**
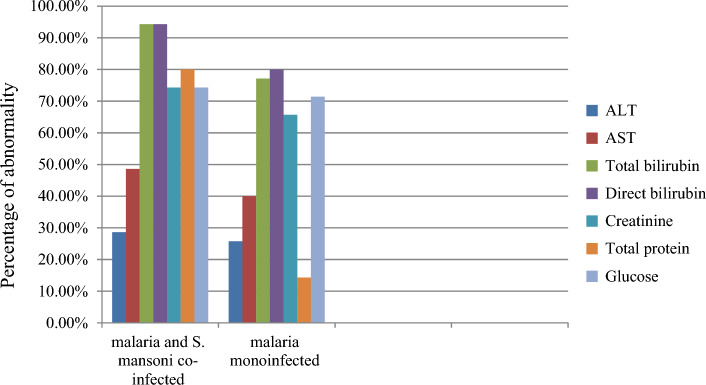
Prevalence of abnormal biochemical profiles of study participants at Dembiya selected health institutions, 2022.

Among malaria and *S. mansoni* co-infected participants, 10 (28.6%), 17 (48.6%), 33 (94.3%), 33 (94.3%), and 26 (74.3%) had elevated ALT, AST, total bilirubin, direct bilirubin, and creatinine, respectively. Similarly, 25 (71.4%), 17 (51.4%), 2 (5.7%), 2 (5.7%), 7 (20.0%), 7 (20%), and 8 (22.9%) of the malaria and *S. mansoni* co-infected participants had normal ALT, AST, total bilirubin, direct bilirubin, creatinine, total protein, and glucose, respectively. On the other hand, 28 (80%) and 26 (74.3%) of the malaria and *S. mansoni* co-infected participants had decreased total protein and glucose, respectively. Similarly, 9 (25.7%), 14 (40.0%), 27 (77.1%), 28 (80%) and 23 (65.7%) had elevated ALT, AST, total bilirubin, direct bilirubin, and creatinine, respectively, in malaria infected participants. However, 26 (74.3%), 21 (60%), 8 (22.9%), 7 (20%), 11 (31.4%), 18 (51.4%), and 10 (28.6%) malaria infected participants had normal ALT, AST, total bilirubin, direct bilirubin, creatinine, total protein, and glucose, respectively. Besides, 5 (14.3%) and 25 (71.4%) of the malaria infected participants had decreased total protein and glucose, respectively, (Fig. 1). Among coinfected male individuals 27.8%, 33.3%, 94.4%, 66.7%, and 88.9% had high ALT, AST, total bilirubin, creatinine, and direct bilrubin, respectively. Also, 29.4%, 64.7%, 94.3%, 82.4, and 100% co-infected females individuals had high ALT, AST, total bilirubin, creatinine, and direct bilrubin, respectively. Additionally, the percentages of elevated ALT, AST, total bilirubin, direct bilirubin, and creatinine in the age 5–14 year co-infected individuals were higher than other age groups. Also, the percentages of lowered total protein and glucose were high in the age 5–14 year group (Tables 3 and 4).

**Table 3:** Prevalence of abnormal biochemical profiles of malaria and *S. mansoni* co-infected study participants at Dembiya selected health institutions, 2022.

Profiles mean (SD)		Age					Gender	
		5–14 N	15–24 N	25–34 N	35–44 N	> 44 N	Male N	Female N
ALT	Normal	3	3	4	4	3	7	7
	High	8	4	4	1	0	11	10
AST	Normal	2	7	5	2	2	8	6
	High	9	1	3	3	1	10	11
Creatinine	Low	0	0	2	0	0	1	1
	Normal	0	1	1	2	3	5	2
	High	11	7	5	3	0	12	14
Total bilirubin	Normal	0	4	3	2	1	1	1
	High	11	4	5	3	2	17	16
Direct bilirubin	Normal	0	2	3	1	1	2	1
	High	11	7	5	3	2	16	16
Total protein	Low	9	5	5	3	1	15	13
	Normal	1	2	1	1	1	2	1
	High	1	1	2	1	1	1	3
Glucose	Low	9	5	5	2	1	14	12
	Normal	2	2	2	2	2	3	5
	High	0	1	1	1	0	1	1

*N* number.

**Table 4:** Prevalence of abnormal biochemical profiles of malaria infected study participants at Dembiya selected health institutions, 2022.

Profiles mean (SD)		Age					Gender	
		5–14 N	15–24 N	25–34 N	35–44 N	> 44 N	Male N	Female N
ALT	Normal	1	3	4	3	2	6	6
	High	10	5	4	2	1	12	11
AST	Normal	5	4	3	2	1	7	8
	High	6	4	5	3	2	11	9
Creatinine	Low	0	0	1	1	0	3	2
	Normal	2	3	3	2	1	5	6
	High	9	5	4	2	2	10	9
Total bilirubin	Normal	2	2	3	2	1	6	4
	High	9	6	5	3	2	12	13
Direct bilirubin	Normal	1	3	3	1	2	8	6
	High	10	5	5	4	1	10	11
Total protein	Low	10	5	4	3	1	8	7
	Normal	1	3	3	2	2	6	4
	High	0	0	1	0	0	4	6
Glucose	Low	10	6	6	3	2	12	14
	Normal	1	2	1	2	1	5	1
	High	0	0	1	0	0	1	2

*N* = number.

### Comparison of biochemical profiles among study participants

In the malaria and *S. mansoni* co-infected participants, the mean [SD] values of ALT, AST, creatinine, total bilirubin, direct bilirubin, total protein, and glucose were 37.1 [7.17] IU/L, 46.9 [8.83] IU/L, 1.48 [0.47] mg/dL, 2.27 [0.69] mg/dL, 0.89 [0.54] mg/dL, 4.74 [1.61] g/dL, and 66.6 [14.0] mg/dL, respectively. And also, the mean [SD] of ALT, AST, creatinine, total bilirubin, direct bilirubin, total protein, and glucose were 36.5 [6.79] IU/L, 39.1 [6.76] IU/L, 1.38 [0.4] mg/dL, 1.81 [0.75] mg/dL, 0.58 [0.51] mg/dL, 4.88 [1.36] g/dL, and 74.5 [8.6] mg/dL in the malaria-infected participants, respectively, (Table 5). The biochemical profiles like ALT, AST, creatinine, total bilirubin, direct bilirubin, glucose, and total protein were normally distributed; a parametric test (Independent t test) was used to compare the mean difference of these biochemical profiles between cases and controls. As a result, an independent t test revealed higher mean values for ALT, AST, creatinine, total bilirubin, and direct bilirubin but lower mean value of Total protein and glucose in malaria and *S. mansoni* co-infected participants than in malaria infected participants. The mean of ALT, AST, total bilirubin, direct bilirubin, and creatinine in the age 5–14 year co-infected individuals were higher than. Also, the mean of total protein and glucose were low in the age 5–14 year group compared to other age groups, (Tables 6 and 7).

**Table 5:** Comparison of biochemical profiles among study participants at Dembiya selected health institutions, 2022.

Profiles	Malaria mono-infected participants mean (SD)	Malaria and *S. mansoni* co-infected participants mean (SD)	p value
ALT (IU/L)	36.5 (6.79)	37.1(7.17)	0.736
AST (IU/L)	39.1 (6.76)	46.9 (8.83)	0.049
Creatinine (mg/dL)	1.38 (0.4)	1.48 (0.47)	0.325
Total bilirubin (mg/dL)	1.81 (0.75)	2.27 (0.69)	0.009
Direct bilirubin (mg/dL)	0.58 (0.51)	0.89 (0.54)	0.018
Total protein (g/dL)	4.88 (1.36)	4.74 (1.61)	0.685
Glucose (mg/dL)	74.5 (8.6)	66.6 (14.0)	0.047

**Table 6:** Age, gender, and infection status based comparison of biochemical profiles among malaria and *S. mansoni* co-infected study participants at Dembiya selected health institutions, 2022.

Profiles mean (SD)	Age						Gender		
	5–14	15–24	25–34	35–44	> 44	p-value	Male	Female	p-value
ALT	39 (7)	37 (7.5)	36.9 (7.1)	37.2 (4)	30 (10)	> 0.05	35.9 (8)	38.3 (5.5)	> 0.05
AST	47 (7.3)	39 (8)	40 (8)	41 (4)	32 (13)	> 0.05	39.8 (10)	44.1 (7.1)	> 0.05
Creatinine	1.8(0.2)	1.5 (0.5)	1.4 (0.7)	1.6 (0.6)	1 (0.1)	> 0.05	1.4 (0.5)	0.60 (0.50)	> 0.05
Total bilirubin	2.6 (0.6)	2.1 (0.6)	2.5 (0.5)	1.6 (0.8)	1.8 (0.3)	< 0.05	2.3 (0.8)	12.28 (0.6)	> 0.05
Direct bilirubin	1.2 (0.5)	0.9 (0.4)	0.8 (0.6)	0.6 (0.6)	0.2 (0.06)	< 0.05	0.8 (0.6)	0.94 (0.5)	> 0.05
Total protein	4 (2)	4.8 (0.5)	5 (1)	5 (2)	4.5 (0.03)	> 0.05	4.9 (1.5)	4.61 (1.72)	> 0.05
Glucose	62 (12.6)	68 (15)	66 (11)	68 (17)	73 (19)	> 0.05	65.7 (16)	67.5 (12.2)	> 0.05

**Table 7:** Age, gender, and infection status based comparison of biochemical profiles among malaria infected study participants at Dembiya selected health institutions, 2022.

Profiles mean (SD)	Age						Gender		
	5–14	15–24	25–34	35–44	> 44	p-value	Male	Female	p-value
ALT	44 (7)	34 (6)	40.5 (7)	38.4 (1.6)	34.5 (8)	> 0.05	35.7 (6)	37.3 (7.4)	> 0.05
AST	45 (6.5)	38 (5.7)	43.4 (5.8)	42.2 (0.7)	34.9 (8.7)	> 0.05	38.6 (6)	39.5 (7.4)	> 0.05
Creatinine	1.5 (0.4)	1.3 (0.3)	1 (0.5)	1.4 (0.6)	1.3 (0.50	> 0.05	1.4 (0.5)	1.4 (0.32)	> 0.05
Total bilirubin	2.5 (0.9)	1.6 (0.4)	2.1 (0.9)	2.1 (0.3)	1.7 (0.8)	> 0.05	1.8(0.6)	1.79 (0.86)	> 0.05
Direct bilirubin	0.9 (0.6)	0.5 (0.41)	0.8 (0.6)	0.5 (0.2)	0.5 (0.5)	> 0.05	0.4 (0.4)	0.7 (0.56)	> 0.05
Total protein	3.5 (1.5)	5.5 (1)	4 (1.4)	5.2 (1.2)	4.5 (0.5)	> 0.05	5.2 (1.3)	4.5 (1.3)	> 0.05
Glucose	60.3 (9.53)	69.7 (5.83)	63.6 (11)	69.1 (2)	72.5 (12)	> 0.05	70 (7.4)	67 (11.4)	< 0.05

### Coagulation profiles and platelet count among study participants

The percentages of prolonged PT, INR, APTT, and low platelet count were higher in malaria and *S. mansoni* co-infected participants than in malaria infected participants.

**Figure 2:**
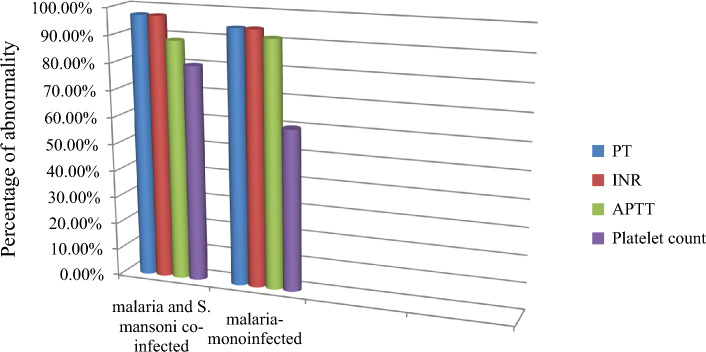
Prevalence of abnormal coagulation profiles and platelet count of study participants at Dembiya selected health institutions, 2022.

In malaria and *S. mansoni* co-infected participants, 34 (97.1%), 34 (97.1%), and 31 (88.6%) had prolonged PT, INR, and APTT, respectively. On the other hand, 1 (2.9%), 1 (2.9%), 1 (2.9%), and 7 (20%) of the malaria and *S. mansoni* co-infected participants had normal values of PT, INR, APTT, and platelet count, respectively. However, 28 (80%) of the malaria and *S. mansoni* co-infected participants had a low platelet count. In malaria infected participants, 33 (94.3%), 33 (94.3%) and 32 (91.4%) had prolonged PT, INR, and APTT, respectively. Likewise, 0 (0%), 1 (2.9%), 3 (8.6%), and 14 (40%) of the malaria infected participants had normal values of PT, INR, APTT, and platelet count, respectively. In addition, only 21 (60%) of the malaria infected participants had a low platelet count (Fig. 2). The percentages of prolonged PT, APTT, and INR in the age 5–14 co-infected individuals were higher than in other age groups. Also, the percentages of low platelets count were high in the age 5–14 year group (Tables 8 and 9).

**Table 8:** Prevalence of abnormal coagulation profiles and platelet count of malaria and *S. mansoni* co-infected study participants at Dembiya selected health institutions, 2022.

Profiles mean (SD)		Age					Gender	
		5–14 N	15–24 N	25–34 N	35–44 N	> 44N	Male N	Female N
PT	Short	0	1	0	1	1	0	0
	Normal	2	1	2	1	2	0	1
	Prolonged	9	6	6	3	0	18	16
INR	Short	1	1	1	0	0	0	1
	Normal	2	2	2	1	1	2	1
	Prolonged	8	5	5	4	2	16	15
APTT	Short	0	1	2	1	0	1	0
	Normal	2	3	2	2	1	7	8
	Prolonged	9	4	4	2	2	10	9
Platelet count	Low	9	4	1	1	1	15	11
	Normal	2	4	7	3	2	3	5
	High	0	0	0	0	0	0	1

*N* number.

**Table 9:** Prevalence of abnormal coagulation profiles and platelet count of malaria infected study participants at Dembiya selected health institutions, 2022.

Profiles mean (SD)		Age					Gender	
		5–14 N	15–24 N	25–34 N	35–44 N	> 44 N	Male N	Female N
PT	Short	1	1	1	0	1	2	1
	Normal	2	3	2	2	2	1	2
	Prolonged	8	4	5	3	0	15	14
INR	Short	0	0	0	0	0	1	1
	Normal	2	4	1	2	1	2	3
	Prolonged	9	4	7	3	2	15	13
APTT	Short	0	2	3	1	0	1	1
	Normal	2	3	2	1	2	3	2
	Prolonged	9	3	3	4	1	14	14
Platelet count	Low	8	5	5	2	1	13	10
	Normal	2	2	2	3	2	5	6
	High	1	1	1	0	0	0	1

*N* number.

### Comparison of coagulation profiles and platelet count among study participants

In the malaria and *S. mansoni* co-infected participants, the mean [SD] values of PT, INR, APTT, and platelet count were 25.3 (5.9) s, 2.43 (0.6), 42.8 (9.9) s, and 127.0 (33.9) × 10^3^/μL, respectively. In addition, the mean [SD] values of PT, INR, APTT, and platelet count were 21.7 [10.20], 2.01 [1.19], 40.6 [5.7] sec, and 140 [64] × 10^3^/μL, respectively, in the malaria infected participants (Table 10).

**Table 10:** Comparison of coagulation profiles and platelet count among study participants at Dembiya selected health institutions, 2022.

Profiles	Malaria mono-infected participants	malaria and *S. mansoni* co-infected participants	p value
PT	21.7 (10.20)	25.3 (5.9)	0.048
INR	2.01 (1.19)	2.43 (0.6)	0.150
APTT	40.6 (5.7)	42.8 (9.9)	0.848
Platelet count (10^3^/μl)	140 (64)	127.0 (33.9)	0.043

The coagulation profiles like PT, INR, APTT, and platelet count were normally distributed; a parametric test (Independent t test) was used to compare the mean difference of these coagulation profiles between cases and controls. As a result, an independent t test revealed higher mean values for PT, INR, and APTT but a lower mean value of platelet count in malaria and *S. mansoni* co-infected participants than in malaria infected participants. The mean of PT, INR, and APTT in the age 5–14 year co-infected individuals were higher than other age groups. Also, the mean of platelet count was low in the age 5–14 year group compared to other age groups (Tables 11 and 12).

**Table 11:** Age, gender, and infection status based comparison of coagulation profiles and platelet count among malaria and S. mansoni co-infected study participants at Dembiya selected health institutions, 2022.

Profiles mean (SD)	Age						Gender		
	5–14	15–24	25–34	35–44	> 44	p-value	Male	Female	p-value
PT	27.5 (5)	20.6 (6.7)	26.1 (5.5)	27.0 (3.98)	23.9 (5.3)	> 0.05	26.2 (5.7)	24.4 (6.1)	> 0.05
INR	2.7 (0.6)	1.9 (0.7)	2.5 (0.6)	2.6 (0.4)	2.2 (0.6)	> 0.05	2.52 (0.61)	2.33 (0.66)	> 0.05
APTT	46 (6.4)	41 (10)	43 (13)	41 (6)	35 (10)	> 0.05	42.7 (10.85)	42.8 (9.01)	> 0.05
Platelet count	101.7 (31)	140 (33)	135 (22)	136 (41)	147 (4)	< 0.05	124.2 (29.9)	129.9 (38.4)	> 0.05

**Table 12:** Age, gender, and infection status based comparison of coagulation profiles and platelet count among malaria infected study participants at Dembiya selected health institutions, 2022.

Profiles mean (SD)	Age						Gender		
	5–14	15–24	25–34	35–44	> 44	p-value	Male	Female	p-value
PT	27.6 (10.5)	22 (5.9)	25.9 (6)	22.7 (4)	21.3 (6)	> 0.05	3.1 (5.8)	23.2 (8.4)	< 0.05
INR	2.8 (1)	2 (0.7)	2.4 (0.70)	2.2 (0.4)	1.9 (0.7)	> 0.05	2.2 (0.59)	2.2 (0.9)	< 0.05
APTT	47 (10.5)	41 (5.3)	45 (7)	46.4 (2)	40 (8)	> 0.05	42.0 (7.3)	42.7 (7.9)	> 0.05
Platelet count	150 (41)	160 (35)	186 (38)	207 (68)	193 (33)	> 0.05	135.9 (30.1)	137.6 (53.4)	< 0.05

## Discussion

Malaria and schistosomiasis co-infections are common, especially in Africa^40^. The co-infection of malaria and schistosomiasis has a significant impact, including exacerbated health consequences and co-morbidities.

In this study, the mean of malaria parasitemia was higher in malaria and *S*. *mansoni* co-infection compared to malaria infection. This finding was similar to a study conducted in Western Ethiopia in 2016 by Mebrate Dufera, that found the mean of malaria parasitemia was higher in malaria and *S*. *mansoni* co-infection compared to malaria mono-infection^41^. However, this finding was in deviation from a study conducted in Northwest Ethiopia which found higher mean malaria parasitemia in malaria monoinfection than malaria and *S*. *mansoni* co-infection^42^. The variation could be explained by variation in the intensity of schistosomiasis co-infection, exposure levels, and duration, and immunity status of the study participants, which would affect malaria parasitemia.

Also, in this study the percentages of elevated ALT, AST, total bilirubin , direct bilirubin, and creatinine in the age 5–14 year malaria and *S*. *mansoni* co-infected individuals were higher than other age groups (15–24 years, 25–34 years, 35–44 years, and > 44 years). In addition, the percentages of lowered total protein and glucose were high in the age 5–14 year group compared to other age groups (15–24 years, 25–34 years, 35–44 years, and > 44 years). This might be due to children's immune systems not be strong enough to combat infections, which makes them more susceptible to develop repeated and potentially fatal infections.

Moreover, in this study mean values of ALT, AST, creatinine, total bilirubin, and direct bilirubin in the malaria and *S. mansoni* co-infected participants were higher than in malaria infected participants. However, mean value of glucose and total protein in malaria and *S. mansoni* co-infected participants were lower than in malaria monoinfected participants. Furthermore, only mean values of AST, total bilirubin, and direct bilirubin were significantly higher but mean value of glucose significantly lower in the malaria and *S. mansoni* co-infected participants than in malaria infected participants. This finding was in consistent with a study conducted in Ethiopia that found lower mean value of glucose and total protein the malaria and *S. mansoni* co-infected participants as compared to malaria infected participants ^42^. This might be as a result of co-infection with *S. mansoni* and malaria, which can result in more severe and chronically devastating morbidity than a parasite infection alone. Participants with co-infections with malaria and *S. mansoni* can suffer from a high amount of intravascular hemolysis of parasitized red blood cells as a result of this, which results in a high level of bilirubin^43^. Additionally, this imbalance in the biochemical profiles may be caused by liver damage brought on by the co-infection of *S. mansoni* and malaria. Additionally, *S. mansoni* and malaria may result in hepatomegaly and jaundice, which raise liver enzymes and bilirubin levels^25,44,45^. Low levels of glucose and total protein could be caused by *S. mansoni* and malaria co-infection's decreased protein synthesis and glucose utilization^46^. On the contrary, this finding was in disagreement with a study conducted in Ethiopia that found higher mean value of ALT and AST the malaria infected participants as compared to malaria and *S. mansoni* co-infected participants. And also, This finding was contradictory with a study conducted in Western Kenya that found significantly lower median value of ALT and creatinine in the malaria and *S. mansoni* co-infected participants as compared to malaria mono-infected participants^47^.

This difference could be brought about by the differences of study participants, exposure levels and duration, infection severity, nutritional status, demographic factors, concurrent infections, parasite strains, and immunity levels.

Furthermore, in this study the percentages of prolonged PT, APTT, and INR in the age 5–14 malaria and *S. mansoni* co-infected individuals were higher than other age groups (15–24 years, 25–34 years, 35–44 years, and > 44 years). Also, the percentages of low platelets count were high in the age 5–14 year group compared to other age groups (15–24 years, 25–34 years, 35–44 years, and > 44 years). This may be because children's immune systems are less strong and matured, making them more vulnerable to infectious diseases and progress to severe disease.

This study showed that PT, INR, and APTT were higher in malaria and *S. mansoni* co-infected participants than in malaria infected participants. However, merely mean values of PT were significantly higher in the malaria and *S. mansoni* co-infected participants than in malaria monoinfected participants. Therefore, it might be concluded that the interaction between *S. mansoni* and malaria may make the coagulation problem worse. This could be as a result of having both malaria and S. *mansoni* infections at the same time, which had considerably higher rates of liver size enlargement than individuals who just had one of the diseases^48^.

In addition to producing cytokines and activating the coagulation system, malaria sequesters red blood cells in the microcirculation. Therefore, red blood cells infected with the malaria parasite stimulate the expression of tissue factors by endothelial cells and assist the formation of coagulation complexes, the production of thrombin, the activation of platelets, the production of microthrombi, and the activation of the intrinsic pathway of coagulation^49^. *Schistosoma mansoni* contributes to coagulation disorders by reducing hepatic production of coagulation proteins and decreasing clearance of activated forms related to coagulation factor consumption^50,51^*.*

In this study, the mean value of platelet count was significantly lower in malaria and *S. mansoni* co-infected participants compared to malaria infected participants. This finding was similar with a study conducted in western Kenya that found a significantly lower median value of platelet count in the malaria and *S. mansoni* co-infected group as compared to the malaria infected group^47^. In cases of malaria and *S. mansoni* coinfection, the decrease in platelet count may be brought on by the consumption and destruction of platelets by the parasites. Since malaria antigens and IgG and IgM antibodies found in immune complexes induce platelets to be trapped by macrophages in the spleen. Due to increased macrophage activity during malaria infection, this results in the loss of more platelets and a reduction in platelet lifespan^52,53^. Additionally, splenomegaly, which results in platelet degradation and filtering by the spleen and thrombocytopenia, may be associated with schistosomiasis^24^. There were limitations on this study. Pregnant women and children under five years old were not allowed to participate in this study; only individuals five years of age and older were participated. Furthermore, we solely used microscopy to measure parasite density; we did not count parasite infections using molecular methods like polymerase chain reaction.

## Conclusions and recommendations

In the current study, biochemical and coagulation profiles, as well as platelet count, in malaria and *S. mansoni* co-infected participants were changed compared to malaria infected participants. Since mean values of ALT, AST, total bilirubin, direct bilirubin, and creatinine were higher, but glucose and total protein were lower in malaria and *S. mansoni* co-infected participants compared to malaria infected participants. Moreover, the mean values of PT, INR, and APTT were higher, but the platelet count was lower in malaria and *S. mansoni* co-infected participants compared to malaria infected participants. This implies that screening patients for biochemical and coagulation profiles, and platelet count changes has greatest role in treating malaria and *S. mansoni* co-infected patients. Furthermore, assessing biochemical and coagulation profiles and platelet count changes in patients with malaria and *S. mansoni* co-infection is an important step toward reducing malaria and *S. mansoni* co-infection associated morbidity and mortality.

Further study need to be conducted to elucidate the possible alteration of biochemical and coagulation profiles and platelet count consequences of malaria and *S. mansoni* co-infection in different epidemiological settings which includes children under the age of 5 years and pregnant women**.** Finally, we would like to recommend that patients be screened for malaria and *S. mansoni* co-infection-associated biochemical and coagulation profiles, and platelet count abnormalities to prevent biochemical and coagulation disorders.

## Data Availability

We confirmed that all the data for this manuscript are available, if someone wants to request the data can contact the corresponding author Mr. Wagaw Abebe.

## Abbreviations

ALT: Alanine aminotransferase
APTT: Activated partial thromboplastin time
AST: Aspartate aminotransferase
EPG: Eggs per gram
INR: International normalized ration
ML: Milliliters
PI: Principal investigator
PT: Prothrombin time
SD: Standard deviation
