# Author Correction: Status of selected biochemical and coagulation profiles and platelet count in malaria and malaria-*Schistosoma mansoni* co-infection among patients attending at Dembiya selected Health Institutions, Northwest Ethiopia

**DOI:** 10.1038/s41598-024-64155-9

**Published:** 2024-06-12

**Authors:** Wagaw Abebe, Zelalem Asmare, Addis Wondmagegn, Mulat Awoke, Aderajew Adgo, Adane Derso, Wossenseged Lemma

**Affiliations:** 1 Department of Medical Laboratory Sciences, College of Health Sciences, Woldia University, Woldia, Ethiopia; 2 Department of Nursing, College of Health Sciences, Woldia University, Woldia, Ethiopia; 3 Department of Biotechnology, College of Natural and Computational Science, Woldia University, Woldia, Ethiopia; 4 Department of Medical Parasitology, School of Biomedical and Laboratory Sciences, College of Medicine and Health Sciences, University of Gondar, Gondar, Ethiopia

Correction to: *Scientific Reports* 10.1038/s41598-024-56529-w, published online 13 March 2024

Adane Derso and Wossenseged Lemma were omitted from the author list in the original version of this Article.

The Author Contributions section now reads:

“W.A. conceived, designed the study, interpretation of the data, and wrote the original draft of the manuscript. Z. A. participated in the data collection and entering data into the software. A.W. participated in the laboratory assay. M. A. participated in the data collection. A. A. performed statistical analysis of the data. W.L. and A.D. were involved in supervising the project, editing, and reviewing the draft of the manuscript. Finally, all the authors read and approved the final manuscript.”

The original Article has been corrected.

